# Arum Triphyllum and Ailanthus Compared

**Published:** 1884-02

**Authors:** C. Carleton Smith

**Affiliations:** Philadelphia


### ARUM TRIPHYLLUM AND AILANTHUS COMPARED.

C. Carleton Smith, M. D., Philadelphia.

| Arum Triph. | Ailanthus. |
| --- | --- |
| **Mind and Sensorium.** | |
| Great irritability. Delirium, tosses and picks his lips or finger nails; bores into the nose; dizziness with fullness of head; sleepy but less stupid than Ailanth. | Low-spirited, decided depression; continuous delirium, muttering, becomes insensible. Confusion of mind with vertigo, intoxicated feeling. |
| **Head.** | |
| Pain in head with vanishing of thought; shooting pains through head. | Headache with dull sensation; cannot bear to think; ideas confused; inclined to be drowsy; face red and often hot. |
| **Eyes.** | |
| As if a veil was let down over them; eyes heavy, sleepy looking; lower lids feel heavy. Smarting of eyes on account of lachrymation; lids on their edges puffy and swollen. | Letters blur and dance up and down; eyes full of tears; looks frightened when aroused; dilation of pupils; smarting and burning in eyes, with secretion of pus. |
| **Nose, Mouth, and Throat.** | |
| Though the nostrils feel dry and stopped, yet there is a free acrid discharge, making the alæ and lips sore and raw, cracked open and bleeding. Interior of mouth so sore that though the child is very thirsty he refuses to drink, and will even cry if the vessel is brought near him.<br>Tongue cracked, burning; prominent papillæ; child dreads to open mouth to have tongue examined.<br>Feeling of constriction of throat with swelling of larynx; can neither chew nor swallow. Much more burning than in Ailanthus. | Copious discharge from nose, ichorous, blood and pus combined. Lips cracked open, forming ragged ulcers, especially near corners of mouth; sordes of teeth quite decided.<br>Astringent feeling in fauces; soreness, worse inhaling cold air; pains extend into ears. Livid swelling of throat and tonsils, with deep ulcers out of which fœtid matter oozes. Swelling of neck with tenderness. |
| **Stomach and Abdomen.** | |
| Complete loss of appetite; burning in œsophagus and stomach; coffee brings on headache; nausea with cramps.<br>Pain in region of liver from front to back. | Food is repulsive; goneness in stomach; sudden violent vomiting on sitting up; stomach feels empty, becomes so inactive it fails to contract. Region of liver quite tender. |
| **Rectum and Stool.** | |
| Soft stools, but with straining; stools yellowish brown and watery, with burning at anus. | Stool quite frequent and painful. Dysenteric in its nature, blood and mucus. Abdomen tympanitic<br>Stools watery, coming out with much force; burning in bowels with weak feeling. |

### Urine.

| Arum Triph. | Ailanthus. |
|---|---|

In both drugs we have suppression, but in Ailanthus the urine is passed unconsciously.

### Larynx and Chest.

| Arum Triph. | Ailanthus. |
|---|---|
| Voice uncontrollable, gives out in singing.<br>Severe cough, very dry; larynx pains continually; larynx sensitive, especially after northwest winds.<br>Rawness with burning from chest to stomach. | Dry hacking cough with feeling as chest would not expand, as if air cells were glued together.<br>Lungs very tender.<br>Burning deep within the chest. |

### Neck and Back.

| Arum Triph. | Ailanthus. |
|---|---|
| Region of atlas very sore and painful; headache, with stiffness of neck. | Dorsal vertebræ all seem to ache; pains in head, neck, and back. |

### Limbs.

| Arum Triph. | Ailanthus. |
|---|---|
| Both hands become swollen and stiff; right leg becomes cramped always on waking out of sleep. Stinging sensation in feet—worse walking. | Tingling or pricking in left arm; both legs feel numb; feeling of tension in feet when walking. |

### Sleep.

| Arum Triph. | Ailanthus. |
|---|---|
| Can't sleep because skin itches so; also sleepless from soreness of mouth; very drowsy, but no signs of stupor. | The great drowsiness soon passes into stupor, preceded by delirium. |

### Fever.

| Arum Triph. | Ailanthus. |
|---|---|
| Frequent chills, with sneezing and yawning. Intense heat of skin, face flushed and burning, especially from 4 to 7 P. M.; desires to escape from bed, and seems unconscious of what he is doing or what is said to him; picks the dry lips all the time until they bleed; bores his nose with finger, and picks the finger-nails; urine very scanty or suppressed; greater nervous excitement than in Ailanthus. | Feels chilly, with sense of hunger and emptiness; after the chill the heat comes on in hot flushes; low form of fever; vomits on rising from recumbent position; face red and hot; first very restless, later drowsy, then muttering and unconscious; teeth filled with sordes; dry tongue, livid or brown down the centre. |

### Skin.

| Arum Triph. | Ailanthus. |
|---|---|
| Eruption resembling scarlet-rash; itching is severe, prevents sleep; skin peels off in patches similar to scarlatina, and has been repeated several times. | Eruption like miliary rash before the chill; generally comes in patches. Between the eruptive points skin is almost livid in color; rash also is of a livid hue; skin becomes torpid, and after passing finger over it the color returns very slowly. |

It is singular, that exposure to cold air under Ailanthus inflames the eyes, while exposure under Arum affects the larynx and produces decided hoarseness.

